# Impact of Particle and Crystallite Size of Ba_0.6_Sr_0.4_TiO_3_ on the Dielectric Properties of BST/P(VDF-TrFE) Composites in Fully Printed Varactors

**DOI:** 10.3390/polym14225027

**Published:** 2022-11-19

**Authors:** Tim P. Mach, Yingfang Ding, Joachim R. Binder

**Affiliations:** IAM-ESS, Karlsruhe Institute of Technology, 76344 Eggenstein-Leopoldshafen, Germany

**Keywords:** inkjet printing, ceramic/polymer composite, tunability, printed varactors, dielectrics

## Abstract

In the field of printed electronics, electronic components such as varactors are of special interest. As ferroelectric materials, Ba_0.6_Sr_0.4_TiO_3_ (BST) and poly(vinylidene fluoride-*co*-trifluoroethylene) (P(VDF-TrFE)) are promising compounds to be used in functional inks for the fabrication of fully inkjet-printed dielectric layers. In BST/P(VDF-TrFE) composite inks, the influence of the particle and crystallite size is investigated by using different grinding media sizes and thermal treatments at varying temperatures. It was found that with an increasing particle and crystallite size, both the relative permittivity and tunability increase as well. However, the thermal treatment which impacts both the particle and crystallite size has a greater effect on the dielectric properties. An additional approach is the reduction in the dielectric layer thickness, which has a significant effect on the maximal tunability. Here, with a thickness of 0.9 µm, a tunability of 29.6% could be achieved in an external electric field of 34 V µm^−1^.

## 1. Introduction

With printed electronics as an emerging technology that employs functional inks to print electrical parts on various substrates, inkjet printing offers the opportunity to produce flexible devices at ambient conditions with a low fabrication cost, a high efficiency in regard to the material consumption and wastage, as well as fast speed with the customizability in design [1,2,3]. Here, functional inks are of special interest, as their performance greatly depends on the properties of the ink components. In this case, non-linear ferroelectrics, whose relative permittivity changes when an external electrical field is applied, can be used in various ways, e.g., for memories [4,5], sensors [6] and electro-optic devices [7]. They are also interesting due to their potential application in the field of tunable microwave devices such as phase shifters [8], filters [9], for antenna beam steering [10] and so on. Here, varactors, which are tunable capacitors, play an important role as key components used in these electric devices [11,12].

One of the most prominent examples for ferroelectric materials is barium strontium titanate (BST), which has been extensively studied. A high permittivity and tunability, a low dielectric loss and the utilization at room temperature makes it a highly desirable candidate for this application [13]. However, ceramics require sintering processes at a high temperature which makes printing on flexible, polymer-based substrates difficult. On the other hand, organic polymers are slowly being recognized as electroactive materials (EAP) and are garnering increasingly more interest in the field of printed electronics. In contrast to inorganic materials, they are light in weight and flexible, albeit displaying lower permittivities and tunabilities. Among these EAPs, poly(vinylidene fluoride)-based polymers, e.g., poly(vinylidene fluoride-*co*-trifluoroethylene) P(VDF-TrFE) are often used, as they exhibit—for polymers—a high ferro-, piezo- and pyroelectricity [13,14,15]. An investigation by Han et al. on P(VDF-TrFE)-based metal–ferroelectric–metal capacitors show that P(VDF-TrFE) as a dielectric material alone, possesses low tunabilities of 2.5% [16]. On the contrary, thin-film BST varactors can reach tunabilites of up to 22–28%, as it was reported by Shen et al. [17]. For bulk materials, Kwon et al. could even obtain high tunabilities of 60% through fine-grained (Ba,Sr)TiO_3_ samples. This can be achieved by controlling the microstructure through the optimization of the sintering conditions and the modification of the composition [18]. Thus, a composite system comprised of inorganic BST and organic P(VDF-TrFE) can be used as a dielectric material to combine their respective advantages. Here, the ceramic particles will function as a particulate system in a polymer matrix to influence the properties of the functional ink. In this regard, studies on the form factor have been conducted for BST/PVDF-based composite systems. The influence of different shapes of the BST powder, i.e., in the form of rods and plates in a BST/PVDF composite system, was investigated by Godziszewski et al. and it was shown that rod-like BST structures display a considerable effect on the tunability, with 9% at 6 V µm^−1^ (@10 GHz) over plate-like structures [19]. In a comparative study with common-sized (Ba_0.55_Sr_0.45_TiO_3_) and nanosized (Ba_0.5_Sr_0.5_TiO_3_) particles in BST-COC composites, Hu et al. observed a higher relative permittivity of 7.3 and a loss tangent of 0.0023 for the nanopowder in contrast to the common-sized BST powder with a relative permittivity of 6.0 and a loss tangent of 0.0009 [20]. For Ba_0.6_Sr_0.4_TiO_3_, Xu et al. observed an increase in the relative permittivity with an increasing sintering temperature and grain size. On the other hand, the dielectric loss displays an initial decrease, followed by an increase with an increasing sintering temperature [21]. Through the variation in the ceramic compound in a solution-casted P-BST/PVDF-based system, Hu et al. showed an increase in the tunability from 3.2% to 7.2% when increasing the BST content from 10 vol% to 40 vol%, with an external electric field of 1 kV/mm (@10 kHz) due to the increase in the relative permittivity [22].

Furthermore, various methods have been employed to manufacture composite films, as shown in Table 1. In a BST/PE (polyethylene) composite system, Hajisaeid et al. employed an extruder in which 34 vol% of BST was mixed with a low-density polyethylene melt to obtain a flexible substrate with a thickness of 600 µm. By printing silver electrodes on top, a varactor system could be attained. At a frequency of 10 GHz, a tunability of 3.5% could be achieved in an electric field of 3 V µm^−1^ [23]. Using a microdispensing printer, Haghzadeh et al. could print BST with a high ceramic content of 80 vol% in a COC-based polymer matrix to realize a microwave varactor. Here, a tunability of 10% (@10 GHz) could be measured with low losses of 0.002 [24]. Wang et al. investigated the influence of the particle size and morphology of plate-like BST in a PVDF matrix. When various particle sizes were fabricated through a two-step molten salt method and through a structural orientation of the BST-plates, a relative permittivity of 62.2 could be measured, which leads to a tunability of 80% at 29 kV mm^−1^ (@1 kHz). The particles were relatively big with 11.47 µm, which made it unsuitable for inkjet printing. Instead, tape casting was used with subsequent hot pressing [25]. Guo et al. conducted a study on the surface-modified BST in BST/P(VDF-TrFE-CTFE) via tape casting with a layer thickness of 100 µm. In this case, with a BST content of 40 vol% in a terpolymer matrix, they could reach a permittivity of 88.3 and reported a tunability of 30% in an electric field of 20 kV mm^−1^ (@1 kHz) [26]. In regard to printing technology, Mikolajek et al. worked on the fabrication of fully inkjet-printed MIM capacitors based on BST/PMMA (poly(methyl methacrylate)) inks. With a ratio of 66.6:33.3, a relative permittivity of 42 (@1 kHz) could be achieved with a layer thickness of 6 µm. Here, it was reported that the relative permittivity of the composite system was about 7 to 18 times higher when compared to pure PMMA [27]. Similar results were yielded by Craton et al. with a BaTiO_3_ (BT)/PVDF system via aerosol jet printing. Through the increase in the BT content, the relative permittivity could be increased. However, at the same time, this also leads to an increase in the dielectric loss. By using aerosol jet printing, layers of 5 µm could be obtained with a maximum capacity of 1.28 pF and a high quality factor of 126 [28]. However, in both works, the tunability was not determined.

Therefore, to the best of our knowledge, inkjet printing has not been utilized to print varactors and tested on their tunability properties yet. In this work, we want to investigate BST/P(VDF-TrFE) as a composite material with the focus on the inorganic BST powder. Through a systematic approach, it was investigated whether the particle and crystallite size have an effect on the dielectric properties and to what degree they influence the tunability and dielectric loss of the BST/P(VDF-TrFE) composite material. By using various grinding media sizes of 200 µm, 400 µm and 800 µm and altering the sintering temperature (700 °C, 900 °C and 1100 °C), the particle size and the crystallite size could be modified. Then, through the addition of P(VDF-TrFE), the composite ink was printed and tested in a fully printed varactor system.

## 2. Materials and Methods

### 2.1. BST Synthesis

Ba_0.6_Sr_0.4_TiO_3_ (BST) was prepared via sol-gel synthesis. Barium acetate (0.422 mol) and strontium acetate hemihydrate (0.281 mol) were dissolved in acetic acid (30.000 mol) under a nitrogen atmosphere. Then, titanium isopropoxide (0.703 mol) was added and after the complexation of titanium alkoxide, the acetic solution was diluted with water. The obtained sol was filtered (PTFE, 1 μm) and spray-dried (MM-HT-ex laboratory spray dryer, Niro, Søborg, Denmark). Afterwards, the precursor was calcined in a tube furnace (HTRH 16/1100/600, Carbolite Gero, Neuhausen, Germany) under synthetic air at 1150 °C for 2 h. The calcined BST powder was milled and dispersed in isopropanol (IPA, ≥99,7, VWR Chemicals, Radnor, PA, USA) using an agitator bead mill (MiniCer, NETZSCH, Selb, Germany) with 200 µm, 400 µm and 800 µm zirconia-grinding media (YTZ, Tosoh, Tokyo, Japan), respectively. The milled BST powder was investigated via inductively coupled plasma-optical emission spectrometry (ICP-OES) where a neglectable amount of Zr (0.926 wt%) from the ZrO_2_ grinding media was found in the BST powder through abrasion during the milling process. The solid content was 14 wt% BST with 0.5 wt% poly(oxy-1,2-ethanediyl), alpha-isotridecyl-omega-hydroxy-, phosphate dispersant (KM3004, Zschimmer und Schwarz, Lahnstein, Germany) as a dispersant. The particle size distribution was measured via laser diffraction (SLS, Horiba LA950, Retsch Technology, Haan, Germany) and the specific surface area (SSA) of the particles was determined with the surface analyzer (Gemini VII 2390a, Micromeritics, Aachen, Germany) via N_2_ physisorption and analyzed by using the multipoint method in accordance with Brunauer, Emmet und Teller (BET). The particle size d_BET_ was calculated using the formular d_BET_ = 6000/(SSA∙ρ_BST_), where ρ_BST_ denotes the theoretical density of BST with 5.682 g cm^−3^ (PDF Card No. 00-034-0411). Prior to the measurements, the samples were dried at 120 °C overnight. The X-ray diffraction (XRD) measurements of the BST powders were performed at a D8 advance A25 diffractometer (Bruker, Billerica, MA, USA, 40 kV, 40 mA) in an angle range of 2*θ* = 15–90° using Cu K*α* (1.5406 Å) with a step size of 0.02°. The measurement time was 1 h and Rietveld refinement was performed using TOPAS. The powders were characterized by using scanning electron microscopy (SEM) (Supra 55, Carl Zeiss, Oberkochen, Germany) with an SE2 detector.

### 2.2. Ink Preparation

#### 2.2.1. Thermal Treatment of BST

The isopropanol of all the suspensions was firstly evaporated at room temperature and the three batches which were milled by different grinding media sizes (200 µm, 400 µm and 800 µm), were divided into three groups (A, B and C, respectively). From each group, one fraction was taken out and assigned with the number 1, i.e., A1, B1 and C1, denoting the samples treated at room temperature. With the exception of group C, the samples of the remaining groups were thermally treated at 700 °C, 900 °C and 1100 °C in a muffle furnace for 2 h with a heating and cooling rate of 5 K/min, and assigned a number from 2 to 4, respectively. For group C, the maximum calcination temperature was 900 °C. A summary of the nomenclature is given in Table 2.

#### 2.2.2. Redispersion of BST

In order to obtain a stable suspension, the three uncalcined BST powders A1, B1 and C1 were redispersed in dimethyl sulfoxide (DMSO, Alfa Aesar, Ward Hill, MA, USA) by using a planetary mill (Pulverisette 7 premium line, Fritsch, Idar-Oberstein, Germany) with a 650 µm zirconia-grinding media (YTZ, Tosoh, Tokyo, Japan) to obtain a 10 vol% BST suspension. For the samples thermally treated at ≥700 °C, the dispersant KM3004 was added again, since the decomposition of the polymer-based dispersant occurs at such high temperatures. For the redispersion, 7 g of BST was mixed with 0.37 g of dispersant and 12.14 g of DMSO by using 70 g of grinding media. The milling time varied between 20 and 50 min. In order to determine the solid content of the BST suspensions, thermogravimetric analysis was performed at 300 °C for 1 h with a heating rate of 5 K/min in a muffle furnace.

#### 2.2.3. Preparation of Composite Ink

The composite inks consist of a BST suspension and a P(VDF-TrFE) (Solvene 200/P200, Sigma Aldrich, Taufkirchen, Germany) solution which have a total solid content of 4 vol% with a volume ratio of BST/P(VDF-TrFE) of 1:1. P(VDF-TrFE) was dissolved in DMSO and methyl ethyl ketone (MEK) in a volume ratio of 1:1 and stirred until dissolution. The solution was then filtered with a PTFE filter (1.0 µm) and mixed with the BST suspension. Additional MEK was further added for the dilution and to adjust the final rheological properties to obtain a final ratio of DMSO/MEK to 1:1. The mixture was ultrasonicated for 15 min. A summary of all the ink compositions is shown in Table 3. For the preparation of the inks, the volumes of the components were converted to masses by using the theoretical densities of the compounds.

The viscosities of the composite inks were measured using a rheometer (Physica MCR 300, Anton Paar, Graz, Austria) with a cone-plate measurement geometry (d_cone_ = 25 mm, α_cone_ = 2°). The measurements were carried out with a controlled shear rate from 1 to 1000 s^−1^ at 30 °C. The surface tension was measured using a force tensiometer with the plate method (K100, Krüss, Hamburg, Germany). The densities were calculated and are between 1.06 and 1.07 g cm^−3^. The viscosity and surface tension were measured for selected inks (A1–A3, B2 and C2). For these inks, the Ohnesorge number was additionally calculated to ensure the printability (Table 4). In accordance with Derby et al., all the prepared inks were within the printable region where the inkjet drop formation was optimized [1].

### 2.3. Printing of Varactors and Dielectric Measurements

The inks were printed on PET substrates (Melinex ST 506 films (175 µm), Dupont Teijin Films, Contern, Luxembourg) using a single nozzle piezoelectric Drop-on-Demand inkjet printer (Autodrop Professional; Microdrop, Norderstedt, Germany). A printhead with 70 μm nozzle diameter was used. A driving voltage between U = 96 V and 128 V, a pulse length between 40 and 54 µs and a printhead temperature between *T* = 17 and 30 °C were used at an ejection frequency of 500 Hz to achieve stable printing conditions. A vacuum of −10 mbar is applied on the ink vessel. The drop formation was monitored using a camera and a strobe diode with a delay time of 500 µs. Drying was done at the substrate table of the printing system, which was heated between 40 °C and 55 °C. The electrodes were printed by using Ag-ink (Silverjet DGP-40LT-15C, Sigma Aldrich, Taufkirchen, Germany) with a printhead using a 50 µm nozzle. The bottom Ag-electrodes were printed on the heated substrate table at a temperature of 80 °C. Then, the sample was sintered at 120 °C for 1 h to make it conductive. Afterwards the dielectric layer was printed on top. Finally, the top Ag-electrode was printed with a substrate temperature of 80 °C and the varactor was dried in a vacuum drying oven at 90 °C for 20 h. The 3D topographies of the printed structures were investigated using a chromatic white light interferometer (MicroProf, Fries Research & Technology, Bergisch Gladbach, Germany). The x–y resolution was 20 μm. Cross sections of the varactor layers were prepared through a target preparation device (Leica EM TXP, Leica Microsystems, Wetzlar, Germany), using a diamond saw and 5 µm SiC lapping foil with subsequent ion beam milling (Leica EM TIC 3X, Leica Microsystems, Germany) and analyzed via SEM (Supra 55, Carl Zeiss, Oberkochen, Germany) using an SE2 detector. Image analysis was performed via ImageJ [29]. The capacitance and the dielectric loss tangent of the varactor samples were determined by an LCR-Meter (E4980AL, Keysight Technologies, Santa Rosa, CA, USA). The measurement was performed with a DC power supply (E36105B, Keysight Technologies, Santa Rosa, CA, USA) with a gradual increase in the voltage from 0 V to 40 V in 10 V steps at a frequency of 200 kHz at room temperature.

## 3. Results and Discussion

### 3.1. Milling

In order to achieve different particle sizes, after the synthesis of the BST powder, the coarse ceramic was firstly milled in an agitator bead mill with ZrO_2_ grinding media of different sizes (200 µm, 400 µm and 800 µm) to obtain a monodisperse suspension (Figure S1). For the unmilled BST powder, a d_99_ value of 175 µm was measured by laser diffraction. After 1 h of milling time, the particle size distribution was checked and the d_99_ values for the suspensions milled by 800 µm (C), 400 µm (B) and 200 µm (A) ZrO_2_ grinding media, were reduced to 2270 nm, 150 nm, and 115 nm, respectively. The d_99_ value of BST-C is this large due to the existence of a second peak at large particles sizes. This is owed to the lower energy input due to the larger grinding media size of 800 µm while maintaining the same mass. Therefore, some bigger particles remain in the suspension and could not be thoroughly milled (Figure S1b). However, in order to keep the milling time comparable, the suspension was used at it is. Furthermore, the fraction of bigger particles is neglectable as the particle size distribution shown is volume-related. When the grinding media size is further decreased, the particle size is reduced even further.

The corresponding specific surfaces are recorded in Table 5. It can be seen that in comparison to the crude BST with a specific surface area of 5 m^2^/g, the milling process drastically increases the surface area by around 5- to 8-fold with decreasing grinding media sizes and decreases the calculated particle size d_BET_. This observation can also be confirmed when the powders were investigated via SEM (Figure S2). For d_BET_, however, it has to be noted that for the calculation, a spherical particle is assumed which is not in agreement with our particles.

### 3.2. Thermal Treatment and Redispersion

Particle size variation can be achieved through the milling process with different grinding media size. For the alteration in crystallite size, however, the ceramic has to be thermally treated and still display a monodisperse distribution with particles which are small enough to be used for inkjet printing. In order to thermally treat the samples, the isopropanol-based suspensions were firstly evaporated at room temperature and then thermally treated at a temperature of 700 °C, 900 °C and 1100 °C, as some prior tests have shown that with increasing temperature, the crystallinity increases, and the selected temperatures display an adequate variety in crystallite size with a constant lattice constant (see Table S1 and Figure S3). At 500 °C, the crystallite size does not differ much from the RT sample. However, with higher temperatures the ceramic particles undergo a sintering process and therefore increase the crystallite size. The challenge lies in the redispersion process of these thermally treated BST powders. Due to the sintering process, agglomeration occurs and have to be disintegrated. However, rigorous milling puts too much stress on the particles and would therefore destroy the enhanced crystallinity of the particles. Hence, after the thermal treatment of the ceramic particles, they have to be redispersed and deagglomerated in a mild manner. This was achieved through utilization of the planetary mill at low speed and a ZrO_2_ grinding media size of 650 µm. Here, the powders were redispersed and deagglomerated in dimethyl sulfoxide (DMSO) and the redispersion process was periodically checked, until a monodisperse distribution or a particle size distribution comparable to the original suspension could be observed via laser diffraction as it is shown exemplarily in Figures S4 and S5.

The specific surface areas with the corresponding d_BET_ values as well as the crystallite sizes and the lattice constants of the BST powders after the thermal treatment and after the redispersion process were measured, respectively (Table 6).

**Figure 1:**
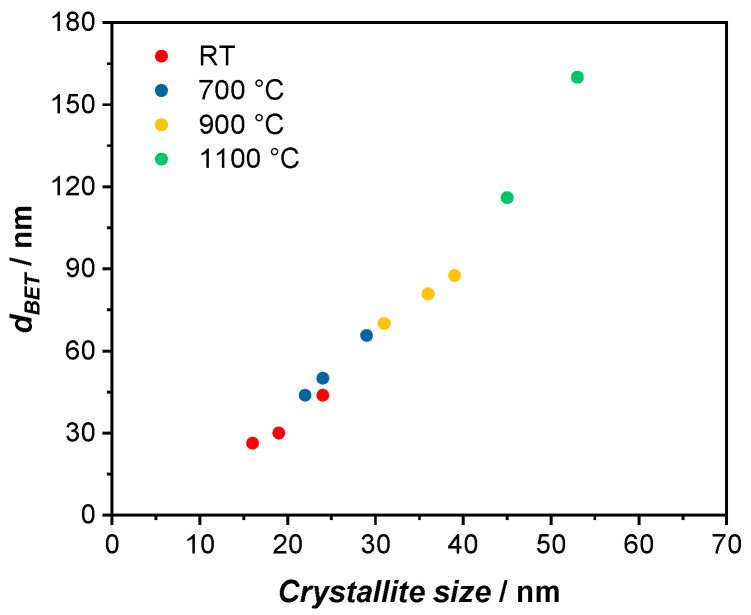
Depiction of the linear dependency of the particle size d_BET_ and the crystallite size at varying temperature. With higher sintering temperature, both the particle size and the crystallite size increase.

By comparing these results, the redispersion process can be controlled, as a higher specific surface area or smaller particle size would imply that the particles were milled and not solely deagglomerated. The same goes for the crystallite size as a smaller crystallite size shows the destruction of the crystalline structures through the milling process. The specific surface area of the thermally treated BST powder decreases with increasing temperature for each group due to the sintering process in which both d_BET_ and the crystallite size increase. Looking at the particle size, the change from RT to higher temperatures is significant. For the A group, an increase to 700 °C, almost doubles the particle size and higher temperatures of 900 °C and 1100 °C even lead to particles which are 3–6 times larger. When applying the particle size against the crystallite size, a linear relation can be observed (Figure 1). Furthermore, the variation in temperature plays a more important role than the variation in grinding media size. For example, for the A group after the thermal treatment, the crystallite size increases from 16 nm (A1) to 24 nm (A2) to 34 nm (A3) to 42 nm (A4) with increasing temperature, while the influence of grinding media size is less prominent with the crystallite size changing from 16 nm (A1) to 18 nm (B1) to 23 nm (C1). However, when comparing the results after the thermal treatment and after redispersion, we can see that the particle size as well as the crystallite sizes stay relatively consistent. Therefore, we can show that the particles were successfully deagglomerated. For samples treated at higher temperatures, i.e., A3, A4, and B4, as well as C2 and C3, a decrease in particle size can be observed. This is due to the redispersion process which breaks up the agglomerated particles. Therefore, a higher surface is accessible which in turn lead to a smaller particle size. Furthermore, for the samples which were sintered at a high temperature of 1100 °C, the suspensions were not stable. Thus, when preparing the ink, phase separation could be observed, where the BST particles were sedimenting and could not be printed. Thus, printed varactors of samples A4 and B4 could not be realized and will not be included in the following discussion.

### 3.3. Ink Preparation and Printing of Varactors

**Figure 2:**
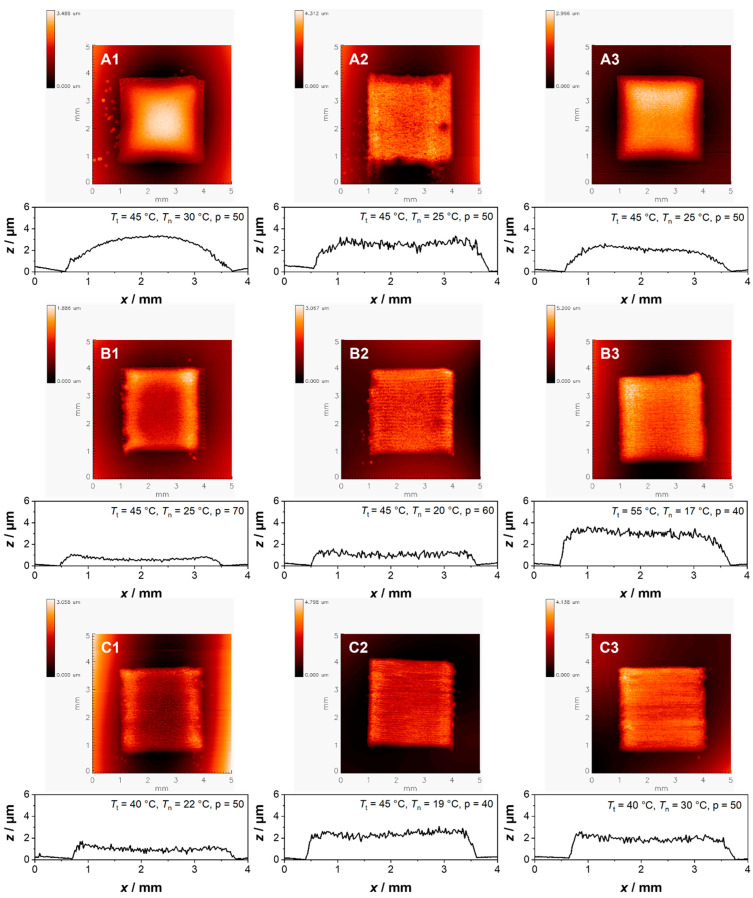
Summary of the topographies and profiles of the printed layers A1–C3, recorded by white light interferometry, where homogeneous layers could be achieved by varying the drying temperature *T_t_*, printhead temperature *T_h_* and the drop spacing *p*.

After the successful redispersion and deagglomeration of the particles, P(VDF-TrFE) was added, and the BST/P(VDF-TrFE) composite ink was used to print the dielectric layer. For the printing process, the variable parameters that could be used to tune the profile and topography of the printed layers, are the temperature of the heating plate *T_t_*, the nozzle *T_n_* and the drop spacing *p*. Through alteration of these parameters, uniform layers could be achieved for each ink as presented in Figure 2. Here, the topography and profiles can be seen, where the coffee stain effect was avoided [30]. The profiles were measured perpendicular to the printing direction and with increasing drop spacing the layers become thinner, as it can be seen for layer B1 and B2 with *p* = 70 and *p* = 60, respectively. In comparison, the other layers were printed with a smaller drop spacing of *p* = 40–50. Interestingly, the roughness of the layer does not relate to the particle size or the applied printing parameters. Looking at the topography, for layer A1–A3, the parameters were the same, yet layer A2 displays a greater roughness when compared to A1 or A3. For layer B1, the topography might indicate a small coffee stain effect, however, the *z*-axis of the profiles is displayed in the µm range while the *x*-axis is in the mm-range. Therefore, the ratio of the profile must be kept in mind, as the coffee stain effect we see here does not affect the uniformity of the layer in a significant way. Furthermore, due to the design of the varactor which will be discussed later in detail, the actual varactors are located in the middle of the printed square. Thus, the quality of the topography is sufficient as long as the middle part of the printed layer is smooth.

For the overall surface quality of the layers, the surface roughness R_a_ was determined which describes the arithmetic average roughness. Based on the recorded data, the values for R_a_ range from 0.079 µm to 0.209 µm (Table 7). For layer A1, A3 and B1, R_a_ is the lowest with 0.079 µm, 0.084 µm and 0.065 µm, respectively, which implies a smoother surface in comparison to the rest, as it can also be seen in Figure 2. Depending on the thickness of the layers, the R_a_ values have a bigger or smaller impact on the overall quality of the printed layer. However, for the thickness measurement as it can be seen in Figure 2, transparent samples, as we have it in our case, pose a challenge for the white light interferometry. Therefore, this method is only to be taken as a first rough determination of the layer thickness [31].

**Figure 3:**
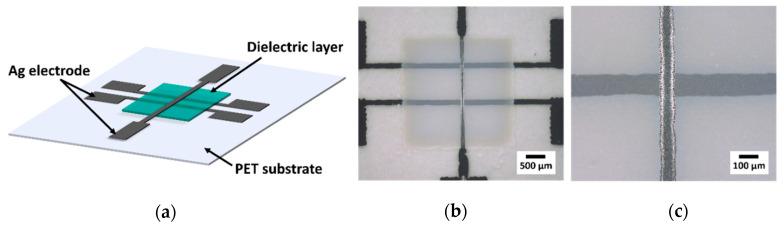
Schematic depiction of (**a**) the printed structure of the varactor on a PET substrate with Ag electrodes and a dielectric layer and (**b**) an optical photo of the printed structure and (**c**) a single varactor.

For a more reliable determination of the layer thickness, SEM images were employed after printing the complete varactor on a PET substrate. The varactor consists of two printed bottom electrodes, a top electrode and the dielectric layer in between (Figure 3a,b). Here, the advantage of this design lies in the utilization of two possible varactors that can be contacted in case the electrodes are not printed well and display disconnected electrode lines. The area of the capacitor can be determined by optical microscopy (Figure 3c) and vary between 11 ∙ 10^3^ µm^2^ and 18 ∙ 10^3^ µm^2^, as shown in Table 7.

**Figure 4:**
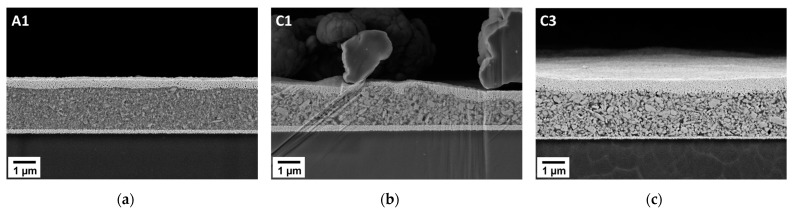
SEM images of the printed varactors (**a**) A1, (**b**) C1 and (**c**) C3 with varying layer thickness and homogeneity through the variation in particle size.

For the thickness determination, a cross section was prepared from a single varactor and measured via SEM. The fully printed varactor is comprised of an Ag electrode at the bottom, followed by the BST/P(VDF-TrFE) dielectric layer and a top Ag electrode as it is exemplarily shown in Figure 4. The obtained thicknesses are in the µm range and the quality of the layers is varying. While a uniform layer thickness could be achieved for varactor A1 (Figure 4a), varactor C1 displays a rougher surface where the thickness could not be easily measured (Figure 4b). For these kinds of samples, an average thickness was calculated by measuring the area of the dielectric layer and dividing it by the length, as it was the case for varactor A2, B2 and C1 (refer to Figure S6). The resulting dielectric layer thicknesses are displayed in Table 7.

For the BST powders that were milled by 800 µm of ZrO_2_ grinding media, a small fraction of larger BST particle sizes is present. Thus, due to this higher degree of inhomogeneity in the particle size, a higher content of large particles can be seen. This is in agreement with the results obtained from the laser diffraction after the milling procedure (Figure S1b). Therefore, the roughness of the dielectric layer also increases as it is shown in Figure 4b,c for varactor C1 and C3, respectively. This phenomenon is even further enhanced due to the thermal treatment which also increases the particle size. For the dielectric layers that were milled by 200 µm of ZrO_2_ grinding media, the observable particles are much finer in comparison, resulting in a smoother profile (Figure 4a). Of course, it should be considered, that the SEM images only provide the cross section of a single image of the dielectric layer. The observed topography is not necessarily representative throughout the whole layer. However, the same must be considered for the profiles obtained by the white light interferometry. While the information obtained from the SEM is on a microscopic level and only represents a restricted two-dimensional area out of the complete dielectric layer, the interferometry method shows the dielectric layer on the macroscopic level. Here, the resolution of 20 µm for the interferometer has to be considered, which means that the measured surface roughness cannot be directly compared to the observations made via the SEM. In addition to the topography, the SEM images show very well the microstructures of the composite layers, which affect the dielectric properties.

### 3.4. Dielectric Properties

After the printing of the varactors, the capacitance and the quality factor of the varactors A1–C3 were measured at room temperature (Table 8). The capacitances of the varactors were all within the pF range, with the highest at 6.21 pF for varactor A2 and the lowest at 1.60 pF for varactor B3. These capacitances are correlated to the thicknesses of the dielectric layer, which are 0.9 µm and 4.1 µm, respectively, and can also be seen in Equation (1), where the capacitance is dependent on the relative permittivity *ε_r_*, the area A and the thickness *d*.

(1) $$C=\varepsilon_{0}\varepsilon_{r}\frac{A}{d}$$

Due to the thin layers in the µm range, the thinner the layer is, the higher the capacitance and vice versa. For the calculation of the quality factor, the reciprocal value of the dielectric loss of tan δ is taken and it can be observed that the attained values are consistently around 20. This equals a tan δ of around 0.05 and does not seem to be dependent on either the thickness, particle size or crystallite size.

**Figure 5:**
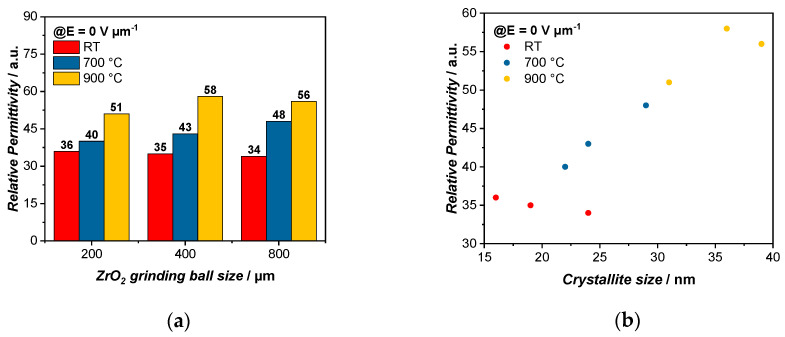
Depiction of the dependency of the relative permittivity ε_r_ on (**a**) the grinding ball size and thermal treatment temperature where the relative permittivity is increasing with higher temperature in each group and (**b**) on the crystallite size, which increases with higher thermal treatment temperature and shows a higher relative permittivity for larger crystallites. The relative permittivities were measured at E = 0 V µm^− 1^.

Through Equation (1), the relative permittivity ε_r_ of the varactor can be calculated (Table 8). When comparing the relative permittivity of the dielectric material at E = 0 V µm^−1^, we can observe an increase in ε_r_ within each group of grinding media sizes A, B and C. As such, for the dielectric BST/P(VDF-TrFE) composite in the A group, the permittivity increases from 36 to 40 to 51 for the samples treated at RT to 700 °C to 900 °C (Figure 5a). A comparable development can be seen for the dielectric layers in the B group and C group, which increase from 35 to 43 to 58 and from 34 to 48 to 56, respectively. However, when comparing the relative permittivity of each temperature group with the varying grinding media size, e.g., A1, B1 and C1, only a small change in ε_r_ is observed. This indicates that the temperature of the thermal treatment, which impacts both d_BET_ and the crystallite size, has a more significant effect on ε_r_, than the particle size through the milling process alone. When plotting the relative permittivity against the crystallite size, a linear behavior can be observed (Figure 5b). With a higher temperature, the crystallite size increases, leading to a larger polarization [32]. Therefore, the relative permittivity increases as well. As we have shown above, the crystallite size correlates with the particle size d_BET_ in a linear fashion (Figure 1). Thus, the relative permittivity also increases with larger particle sizes.

A similar trend can be observed for the measured tunabilities τ of the varactors, which are calculated using Equation (2). Here, *ε_r_*(0) and *ε_r_*(*V*) denote the relative permittivity at a zero and non-zero DC electric field, respectively [33].

(2) $$\tau =\frac{\varepsilon_{r}\left(0\right)-\varepsilon_{r}\left(V\right)}{\varepsilon_{r}\left(0\right)}\;\cdot 100\;\%$$

**Figure 6:**
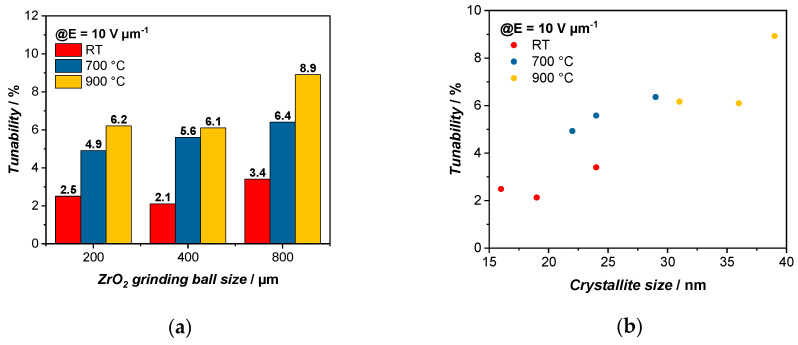
Depiction of the dependency of the tunability τ on (**a**) the grinding media size and thermal treatment temperature. The measured tunabilities increase with higher sintering temperature and display a slightly higher tunability with increasing grinding media sizes. A linear trend can be observed in (**b**) where the tunability is improved with increasing temperature which in turn leads to larger crystallite sizes. The tunabilities were recorded for E = 10 V µm^−1^.

In order to be able to compare the tunabilities, the values were chosen for an electric field of 10 V µm^−1^. It is observed that the tunabilities also increase within the same group of grinding media sizes with a higher temperature from 2.5% to 4.9% to 6.2% for the A group and from 2.1% to 5.6% to 6.1% for the B group and from 3.4% to 6.4% to 8.9% for the C group (Figure 6a). The particle size also influences the tunability of the varactors and it can be seen that with a larger particle size, the tunabilities increase as well, since they have a higher dielectric response, as it is easier to form a percolation path with larger particles [34]. While for the varactors treated at RT, the tunabilities change from 2.5% to 2.1% to 3.4%, at a higher temperature of 900 °C, an increased tunability can be observed from 6.2% to 6.1% to 8.9%. Since with a higher relative permittivity, the tunability also increases, we can see that the tunability improvement is also favored by the increasing temperature rather than the milling process [35]. Here, the same as above, a linear trend can be observed when plotting the tunability against the crystallite size (Figure 6b).

The role of the particle size and crystallite size was investigated by comparing the tunabilities of the varactors at 10 V µm^−1^. However, for the varactors, the electric field strength plays a major role when it comes to the maximum tunability. Since the applied external electric field is dependent on the thickness, the maximum tunability increases even further the thinner the layers are. However, the microstructure of the composite also influences the tunability. When comparing the maximum achievable tunability τ_max_ of varactor A1 and A3, which both have a comparable thickness of 2.3 µm and 2.1 µm, respectively, A1 exhibits a tunability of 5.4% while A3 displays a tunability of 12.2% (Table 8). This is due to the influence of the thermal treatment which results in a larger particle and crystallite size, and therefore the increase in the relative permittivity and tunability. However, when looking at varactor A2, it becomes clear that the thickness of the dielectric layer plays a major role. If the layer thickness is disregarded, the expected tunability should be between A1 and A3. However, due to a very thin layer of 0.9 µm, varactor A2 outperforms all the others and exhibits the highest tunability with 29.6% in an electric field of 34 V µm^−1^ (Figure S7). A major drawback on the other hand is the lower breakdown voltage that comes with thinner layers. Hence, when the maximum DC voltage of 40 V was applied to varactor A2, it broke down and was not usable anymore afterwards.

## 4. Conclusions

BST nanoparticles with a varying particle size and crystallite size were obtained through agitator bead milling with different ZrO_2_ grinding media sizes of 200, 400 and 800 µm and a thermal treatment at room temperature, 700 °C, 900 °C and 1100 °C, respectively. Upon a successful redispersion of the thermally treated BST powders, the ferroelectric polymer P(VDF-TrFE) was added and BST/P(VDF-TrFE) composite inks with different particle sizes, ranging from 25 to 175 nm and crystallite sizes between 16 and 53 nm, were prepared and printed on flexible PET substrates.

Through adjustments of the printing parameters and drying temperature, the coffee stain effect was prevented. However, for the printed layers, which are supposed to be in the µm range, larger particles, especially from the C group, lead to a higher surface roughness. For inkjet printing, there was a limitation on the thermal treatment, as sedimentation processes can be observed in the ink when a sintering temperature of 1100 °C is used. With the printable composite systems, relative permittivities from ε_r_ = 34 up to ε_r_ = 58 could be realized. The effects of the modified BST particles were investigated, and it was found that the relative permittivity and the tunability are linearly correlated with the particle size and crystallite size. Thus, with increasing the particle size, ε_r_ and τ increase. In comparison to the variation in particle size through the milling procedure, the thermal treatment has an even greater impact on the relative permittivity and tunability. Furthermore, it could be shown that the layer thickness plays a major role in the tunability of the varactor as higher field strengths can be achieved. In this work, it is demonstrated that at a dielectric layer thickness of 0.9 µm, the varactor A2 could outperform all the other samples with a maximal tunability of 29.6% in an electric field of 34 V µm^−1^. Therefore, in order to achieve varactors with a high tunability, the optimization of the thermal treatment and thickness of the dielectric layer offer a promising way. However, here, the challenge lies in the printability of the high sintered BST powders and the breakdown voltage for thin layers and high field strength. For these high sintered BST powders, other printing techniques such as microdispensing [36] could be employed.

In summary, we report a fully printed BST/P(VDF-TrFE) composite varactor system with measured tunabilities which, to the best of our knowledge, has not been investigated before. The dielectric properties, such as the tunability and quality factor of the varactors, were fundamentally compared in regard to their particle size and crystallite size at a frequency of 200 kHz. In order to measure the dielectric properties of the varactors in the scope of application, they will be tested in the high frequency region (GHz) as a next step.

## Figures and Tables

**Table 1:** Comparison of the characteristics of BST/polymer-based dielectrics, published in the literature.

Dielectric	Ratio [vol%] (cer./pol.)	Layer Thickness [µm]	Permittivity [a.u.]	Tunability [%]	Dielectric Loss [a.u.]	Fabrication Method	Literature
BST/PE	34	600	16 (@10 GHz)	3.5 (3 V µm^−1^)	0.04	Extruder	[23]
BST/COC	80	60	38 (@10 GHz)	10 (5 V µm^−1^)	0.002	Microdis-pensing printer	[24]
BST/PVDF	40	100	13 (@10 GHz)	9 (6 V µm^−1^)	0.03	Tape casting	[19]
P-BST/PVDF	20	100–200	62.2 (@1 kHz)	80 (29 V µm^−1^)	0.042	Tape casting	[25]
BST/P(VDF-TrFE-CTFE)	0–40	100	53.48–88.32 (@1 kHz)	17–30 (20 V µm^−1^)	0.056 @40 vol%	Tape casting	[26]
BST/P(VDF-TrFE)	50	0.89	40 (@200 kHz)	29.6 (34 V µm^−1^)	0.06	Inkjet printing	This work

**Table 2:** Summary of used nomenclature depending on the grinding media size and thermal treatment.

	Temperature [°C]	RT	700	900	1100
ZrO_2_ Grinding Media Size [µm]					
200		A1	A2	A3	A4
400		B1	B2	B3	B4
800		C1	C2	C3	-

**Table 3:** Summary of the concentration and volume of the ink components.

	Concentration [vol%]		Volume [mL]		
Ink	BST in DMSO	P(VDF-TrFE) in DMSO/MEK (1:1)	BST in DMSO	P(VDF-TrFE) in DMSO/MEK (1:1)	MEK
A1	9.1	3.5	0.56	1.44	0.50
A2	7.7	4.0	1.00	1.30	0.63
A3	7.6	4.1	1.02	1.28	0.65
A4	7.1	4.5	0.78	1.22	0.75
B1	9.4	3.4	0.53	1.47	0.48
B2	7.2	4.3	0.75	1.25	0.69
B3	8.9	3.6	0.57	1.43	0.52
B4	8.1	3.8	0.64	1.36	0.59
C1	9.2	3.4	0.54	1.46	0.50
C2	7.2	4.3	0.75	1.25	0.69
C3	6.6	4.9	0.85	1.15	0.79

**Table 4:** Overview of the fluid mechanical properties of the inks A1–A3, B2 and C2.

Ink	Viscosity [mPa∙s]	Surface Tension [mN m^−1^]	*Oh* Number [-]
A1	9.2	22.2	0.23
A2	6.4	23.7	0.15
A3	6.3	22.6	0.15
B2	6.4	19.5	0.17
C2	6.0	18.7	0.16

**Table 5:** Results of the specific surface area with the calculated d_BET_.

ZrO_2_ Grinding Media Size [µm]	Specific Surface Area [m^2^ g^−1^]	d_BET_ [nm]
Crude BST	5	210
800	26	81
400	38	42
200	42	34

**Table 6:** Comparison of specific surface area, d_BET_, crystallite size and lattice constant of the BST powders after milling, thermal treatment and redispersion. The BST has a cubic structure in all cases.

BST	Specific Surface Area [m^2^ g^−1^]	d_BET_ [nm]	Crystallite Size [nm]	Lattice Constant [Å]
After milling				
A	42	25	16 (1)	3.966
B	38	28	18 (1)	3.966
C	26	40	23 (1)	3.965
After thermal treatment				
A1	42	25	16 (1)	3.966
A2	23	46	24 (1)	3.967
A3	14	75	34 (1)	3.969
A4	7	150	43 (1)	3.971
B1	38	28	18 (1)	3.966
B2	20	53	25 (1)	3.966
B3	13	81	36 (1)	3.968
B4	6	175	53 (1)	3.970
C1	26	40	23 (1)	3.965
C2	14	75	31 (1)	3.965
C3	9	117	41 (1)	3.967
After redispersion				
A1	40	26	16 (1)	3.966
A2	24	44	22 (1)	3.966
A3	15	70	31 (1)	3.970
A4	9	116	45 (1)	3.972
B1	35	30	19 (1)	3.965
B2	21	50	24 (1)	3.966
B3	13	81	36 (1)	3.968
B4	7	160	53 (1)	3.970
C1	24	44	24 (1)	3.965
C2	16	66	29 (1)	3.965
C3	12	88	39 (1)	3.966

**Table 7:** Summary of the surface roughness R_a_, thicknesses and area sizes of the printed layers A1–C3. The thicknesses were determined via SEM and the area sizes via optical microscopy.

Layer	R_a_ [µm]	Thickness Dielectric Layer ^1^ [µm]	Area of the Varactor [10^3^ µm^2^]
A1	0.079	2.31 ± 0.20	13.5
A2	0.208	0.89 ± 0.20	15.8
A3	0.084	2.14 ± 0.20	12.9
B1	0.065	1.07 ± 0.20	17.5
B2	0.150	1.42 ± 0.20	16.0
B3	0.209	4.14 ± 0.20	12.8
C1	0.129	1.42 ± 0.20	11.6
C2	0.198	2.62 ± 0.20	15.6
C3	0.190	2.16 ± 0.20	18.2

^1^ Thickness of the dielectric layers were measured by SEM.

**Table 8:** Summary of the dielectric properties of the varactors with their corresponding capacitance and quality factor as well as the relative permittivity ε_r_, tunabilities @10 V µm^−1^ and maximum tunability of the varactors A1–C3.

Varactor	Capacitance [pF]	Q-Factor [a.u.]	ε_r_ [a.u.]	τ @10 V µm^−1^ [%]	τ_max_ [%]
A1	1.88	20.3	36	2.5	5.4
A2	6.21	16.3	40	4.9	29.6
A3	2.71	19.5	51	6.2	12.2
B1	5.10	20.4	35	2.1	13.9
B2	4.25	18.1	43	5.6	18.1
B3	1.60	20.1	58	6.1	5.6
C1	2.46	19.0	34	3.4	11.4
C2	2.51	20.0	48	6.4	9.2
C3	4.14	21.3	56	8.9	17.2

## Data Availability

Not applicable.

## Supplementary Materials

The following supporting information can be downloaded at: https://www.mdpi.com/article/10.3390/polym14225027/s1, Figure S1. Particle size distribution of (a) the crude BST and the suspensions milled with (b) 800 µm, (c) 400 µm and (d) 200 µm ZrO2 grinding media. Figure S2. SEM images of (a) the crude BST and the suspensions milled with (b) 800 µm, (c) 400 µm and (d) 200 µm ZrO2 grinding media. Table S1. Thermal treatment tests ranging from RT to 500–1100 °C with crystallite sizes and lattice constants for BST particles milled by 400 µm ZrO_2_ grinding media. Figure S3. XRD data of the thermal treatment tests ranging from RT to 500–1100 °C for BST particles milled by 400 µm ZrO_2_ grinding media. Figure S4. Development of the particle size distribution of BST-A1 over time. Figure S5. Development of the particle size distribution of BST-C3 over time. Figure S6. Summary of the cross section of the varactors A1–C3. Figure S7. Depiction of the tunabilities of the varactors dependent on the electric field for the (a) A group, (b) B group and (c) C group.
