# Supplementary material for: Impact of Particle and Crystallite Size of Ba0.6Sr0.4TiO3 on the Dielectric Properties of BST/P(VDF-TrFE) Composites in Fully Printed Varactors
Source: Polymers (Basel). 2022 Nov 19;14(22):5027. doi: 10.3390/polym14225027 (PMC9698761; doi:10.3390/polym14225027)
Supplement: Supplementary file 1 [file polymers-14-05027-s001.zip › polymers-1981373-supplementary.pdf]

polymers

MDPI

Supplementary Materials

Impact of Particle and Crystallite Size of $Ba_{0.6}Sr_{0.4}TiO_3$ on the Dielectric Properties of BST/P(VDF-TrFE) Composites in Fully Printed Varactors

Tim P. Mach *, Yingfang Ding and Joachim R. Binder

IAM-ESS, Karlsruhe Institute of Technology, 76344 Eggenstein-Leopoldshafen, Germany
* Correspondence: tim.mach@kit.edu

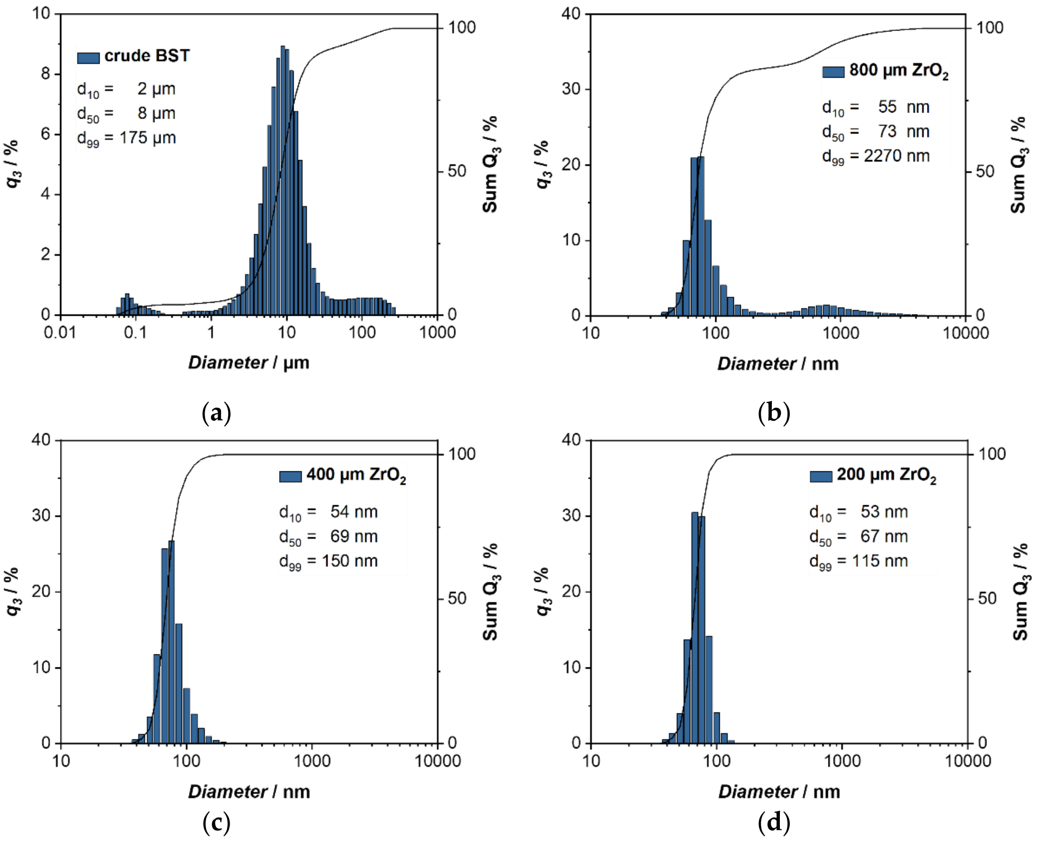

Figure S1. Particle size distribution of (a) the crude BST and the suspensions milled with (b) 800 µm, (c) 400µm and (d) 200 µm ZrO_2 grinding media.

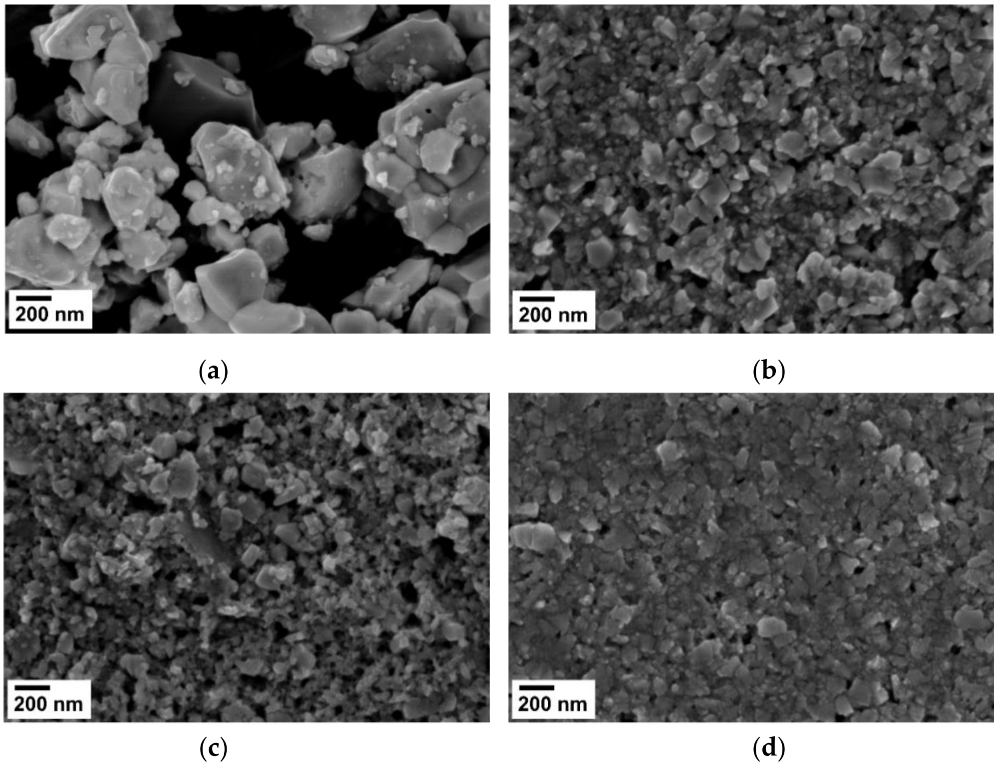

Figure S2. SEM images of (a) the crude BST and the powders milled with (b) 800 µm, (c) 400µm and (d) 200 µm ZrO_2 grinding media.

Table S1. Thermal treatment tests ranging from RT and 500 – 1100 °C with crystallite sizes and lattice constants for BST particles milled by 400 µm ZrO_2 grinding media.

Temperature / °C	Crystallite size / nm	Lattice constant / Å
RT	19 ± 0.2	3.965
500	20 ± 0.1	3.965
600	24 ± 0.2	3.965
700	29 ± 0.2	3.967
800	33 ± 0.3	3.968
900	41 ± 0.5	3.969
1000	53 ± 0.8	3.969
1100	73 ± 1.3	3.970

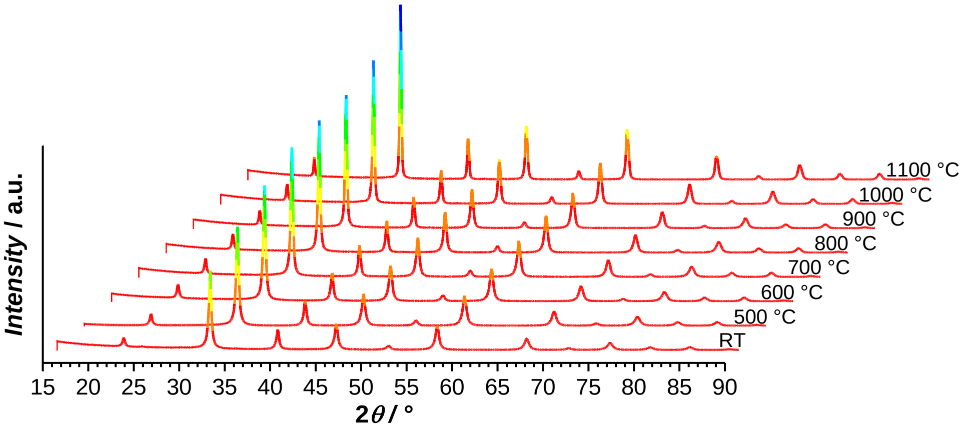

Figure S3. XRD data of the thermal treatment tests ranging from RT and 500 – 1100 °C for BST particles milled by 400 µm ZrO_2 grinding media.

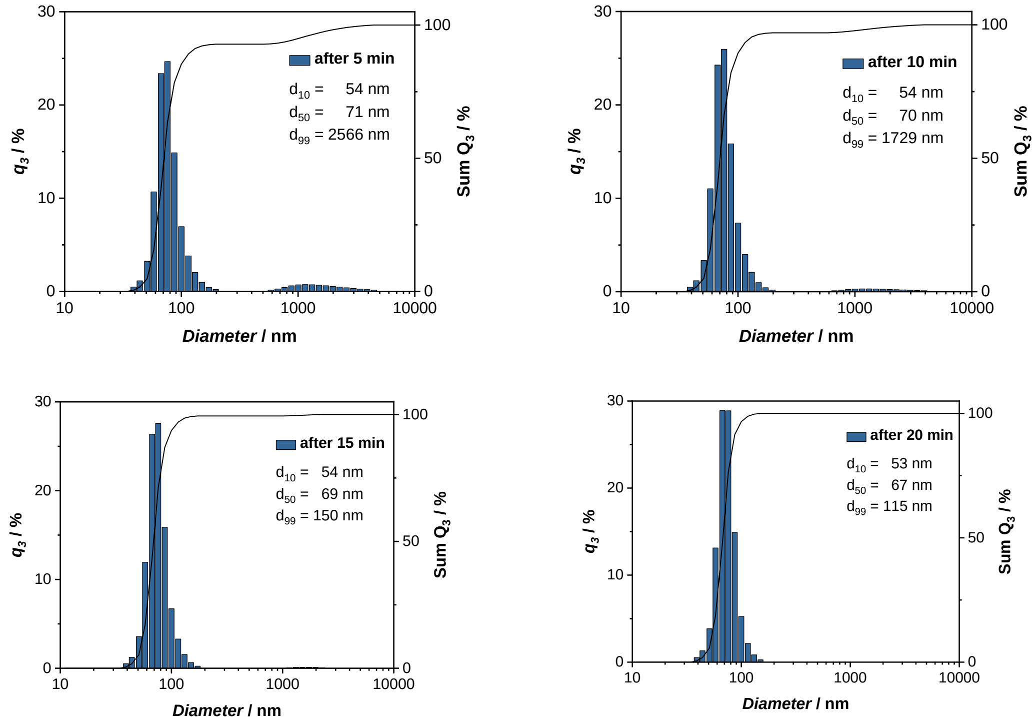

Figure S4. Development of the particle size distribution of BST-A1 over time.

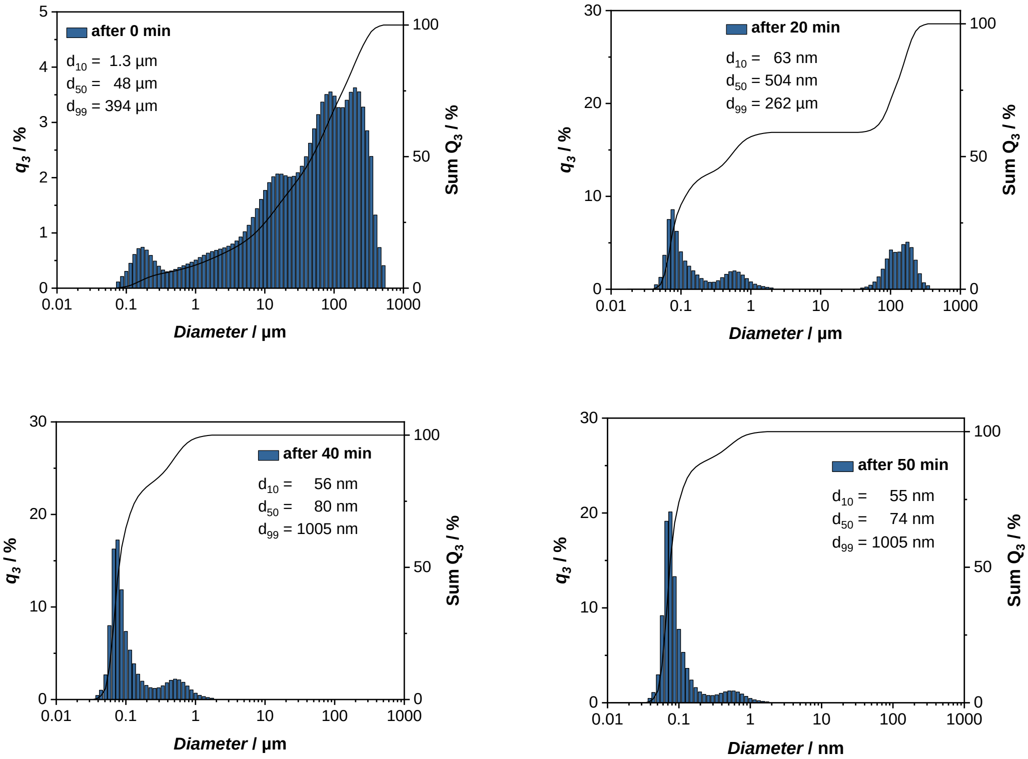

Figure S5. Development of the particle size distribution of BST-C3 over time.

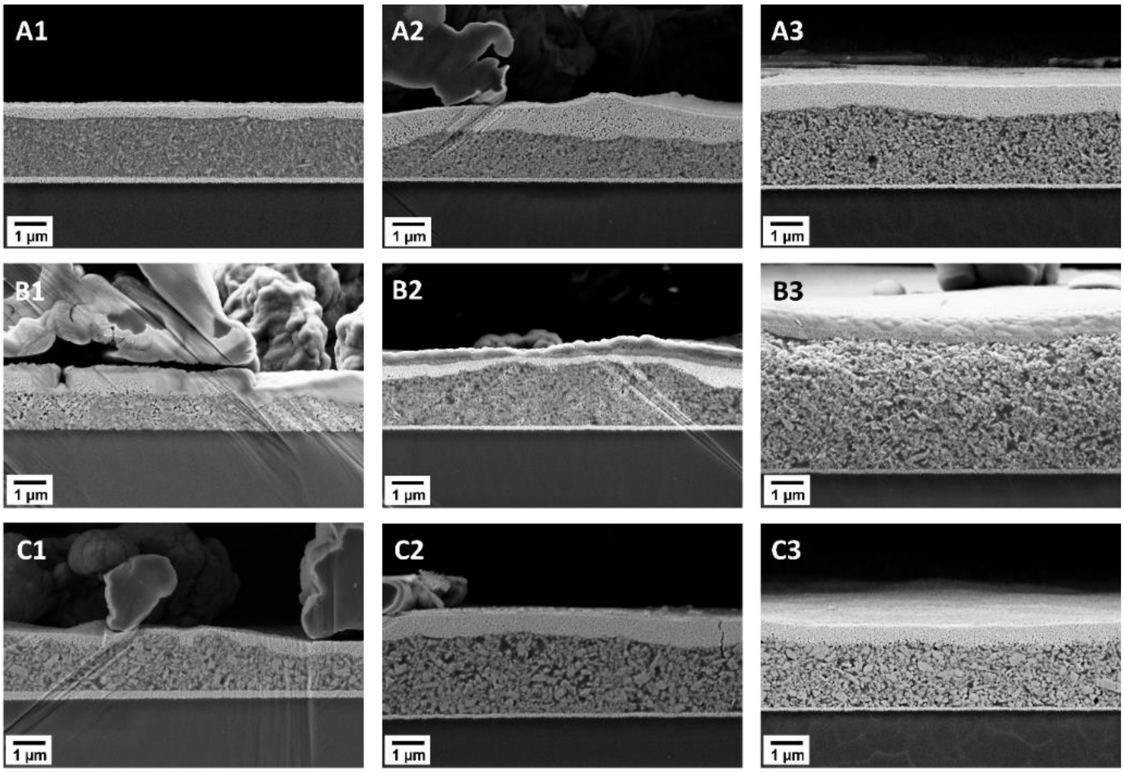

Figure S6. Summary of the cross section of the varactors A1 – C3.

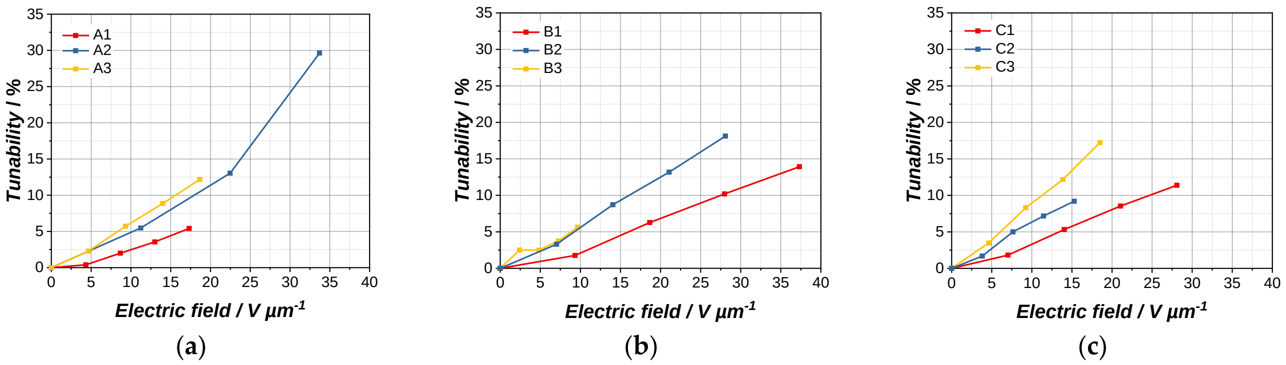

Figure S7. Depiction of the tunabilities of the varactors dependent on the electric field for the (a) A-group, (b) B-group and (c) C-group.
